# Molecular insights into the interaction of HPV-16 E6 variants against MAGI-1 PDZ1 domain

**DOI:** 10.1038/s41598-022-05995-1

**Published:** 2022-02-03

**Authors:** Lilian Esmeralda Araujo-Arcos, Sarita Montaño, Ciresthel Bello-Rios, Olga Lilia Garibay-Cerdenares, Marco Antonio Leyva-Vázquez, Berenice Illades-Aguiar

**Affiliations:** 1 Laboratorio de Biomedicina Molecular, Facultad de Ciencias Químico-Biológicas, Universidad Autonóma de Guerrero, 39090 Chilpancingo, CP México; 2 Laboratorio de Bioinformática y Simulación Molecular, Facultad de Ciencias Químico Biológicas, Universidad Autónoma de Sinaloa, 80030 Culiacán Sinaloa, CP México; 3 CONACyT-Universidad Autónoma de Guerrero, 39087 Chilpancingo, CP México

**Keywords:** Cancer, Computational biology and bioinformatics

## Abstract

Oncogenic protein E6 from Human Papilloma Virus 16 (HPV-16) mediates the degradation of Membrane-associated guanylate kinase with inverted domain structure-1 (MAGI-1), throughout the interaction of its protein binding motif (PBM) with the Discs-large homologous regions 1 (PDZ1) domain of MAG1-1. Generic variation in the E6 gene that translates to changes in the protein’s amino acidic sequence modifies the interaction of E6 with the cellular protein MAGI-1. MAGI-1 is a scaffolding protein found at tight junctions of epithelial cells, where it interacts with a variety of proteins regulating signaling pathways. MAGI-1 is a multidomain protein containing two WW (rsp-domain-9), one guanylate kinase-like, and six PDZ domains. PDZ domains played an important role in the function of MAGI-1 and served as targets for several viral proteins including the HPV-16 E6. The aim of this work was to evaluate, with an in silico approach, employing molecular dynamics simulation and protein–protein docking, the interaction of the intragenic variants E-G350 (L83V), E-C188/G350 (E29Q/L83V), E-A176/G350 (D25N/L83V), E6-AAa (Q14H/H78Y/83V) y E6-AAc (Q14H/I27RH78Y/L83V) and E6-reference of HPV-16 with MAGI-1. We found that variants E-G350, E-C188/G350, E-A176/G350, AAa and AAc increase their affinity to our two models of MAGI-1 compared to E6-reference.

## Introduction

High-risk human papillomaviruses (HR-HPV) are the principal etiological agents of cervical cancer (CC), being the HPV-16 genotype one of the most prevalent worldwide^1^. The encoding proteins E6 and E7 from HPV-16 are the major oncogenic determinants of the disease’s progression. These proteins control regulatory functions of the cell cycle, promote proliferation, induce malignant transformation, and facilitate migration and invasion of transformed cells^2^.

Interestingly, the tumorigenic potential of HPV-16 differs among infected women. It has been proposed that the changes in the amino acidic sequence of E6 are a major risk factor for the development and aggressiveness of the disease^3^. The variants E-G350 (L83V), E-C188/G350 (E29Q/L83V), E-A176/G350 (D25N/L83V), AAa (D25N/L83V), and AAc (Q14H/I27R/H78Y/L83V) were found to be the most prevalent in a population from Guerrero, Mexico, a state with the highest poverty and marginalization rates in the country^4^. Experimental studies suggested that variants differ in their ability to affect several important cellular processes, including differentiation, apoptosis, immortalization, migration and metastasis^3,5–9^. But, the molecular insights about this,are not clear^5,10,11^.

HPV-16 E6 protein has 151 amino acids and structurally it contains two zinc-binding domains with two C–x–x–C motifs each, which are vital for the oncogenic potential of the virus^12^. Furthermore, its sequence harbors a PDZ (PSD95, DLG, and ZO1) class I binding motif (PBM) (E148, T149, Q150, and L151), located at the carboxyl terminus of the protein^13^. Viral proteins often target PDZ domains to induced their degradation, resulting in the disruption of cellular processes that benefits the viral cycle^14^. Accumulated functional evidence suggested that PDZ motif is sufficient for the transformation of primary human keratinocytes, hyperplasia and carcinogenesis in E6-transgenic mice^15–17^.

In contrast of the well documented role of E6 in biological activities, here are only a few biophysical and structural estudies of this protein due to the difficulty in its preparation as a soluble protein. E6 of HPV-16 is difficult to express under a native soluble form in bacteria due to its hight content of cisteins that promotes the formation of inclusion bodies^12^. E6 has been produced fused to Maltose binding protein MBP at its C-terminus, but, it is mainly produced in the form of soluble high molecular weight aggregates^18^. The full-length protein, fused to a His_6_-tagged was also produce in inclusion bodies and refolded^19^. Moreover, His_6_-tagged E6 proteins from HPV16, without any modifications, had been produced in bacteria as soluble and stable molecules and structural analyses suggests that it maintains correct folding and conformational properties^20^. E6 has been produced as an insoluble protein and as a soluble protein that contains mutated cysteins to serines^21^. However in each case, the resulted protein has not been sufficient for its biophysical characterization under native conditions, this limits the knowledge of its interactions with target proteins involved in the tumorigenesis^22^. To our knowledge, the only avalible 3D structure of E6 can be found in a ternary complex comprising full-length human papilloma virus type 16 (HPV-16) E6, the LxxLL motif of E6AP and the core domain of p53^21^. The present crystallized structure constitutes an ideal experimental model for the design and molecular characterization of the interaction of the E6 oncoprotein with different target proteins.

HPV-16 E6 targets multi-PDZ domain protein MAGI-1 a scaffolding protein found at tight junctions of epithelial cells^23–25^. MAGI-1 is a multidomain protein composed of two domains WW (rsp-domain-9) (G300–C333 and L359–L392) a protein–protein binding domain that mediates specific interactions with short proline-rich or proline-containing motifs, one guanylate kinase-like domain (A96-287F), and six PDZ domains located at E17-G105, H472-R554, T643-R721, S813-P895, S970-S1066 and E1124-T1206, that are composed of approximately 80–110 residues^26,27^.

E6 of HPV-16 targets MAGI-1 PDZ1 and in vitro experiments proposed that the degradation of MAGI-1 is mediated by the direct interaction of its PDZ-1 domain (H472-R554) with the PBM motif from E6 (E148-L151)^17,28^. Moreover, the adjacent amino acids of E6 that may play a key role in the interaction of these proteins (C103-I104, R135-C136, C139-S140, S82, G85, L88, S97, N105, R124-F125 and N127-I128)^22,29^. In the case of MAGI-1, amino acids close to the PDZ1 domain are thought to have a role in their interaction too^28–30^. Interestingly, structural analysis of PDZ domains and PDZ-mediated interactions by NMR and X-ray crystallographic methods in conjunction with computational methods had provided insights into the specificity or promiscuity of PDZ protein–protein interactions^28^. Accumulating studies showed, that the binding preferences of a single PDZ domain protein differ from that of a multiple PDZ domain protein or a protein with a combination of domain, therefore, careful examination of the binding properties of proteins containing tandem PDZ domains or PDZ domains combined with other interaction module is required^31^.

E6 of HPV-16 targets many PDZ containing cell proteins, the details of their interactions remains unclear, but, Fournane et al*.,* confirmed that residues located inside and outside the canonical PDZ domain of MAGI-1 and E6 PBM motif are necessary for the interaction of these proteins^30^. Taking into account the experimental proven regions used to stablish the direct interaction of E6 and MAGI-1 that included the PMB motif of E6 and the PDZ-1 domain of MAGI-1^32^, we predict two MAGI-1 models that included the PDZ-1 domain and adjacent regions that were observed to have a role in the interaction of these proteins^13,14,23–25,30,33^. We used these models to get insights into the of the E6 variants with MAGI-1.

Recently, our team has adopted an in-silico analysis approach to evaluate the structural changes of E6 and its variants^34,35^. Moreover, we are interested in the prediction of the interaction of E6 variants with the PDZ-1 domain of MAGI-1.

## Results and discussion

HPV-16 is accounting for more than 70% of the CC cases^1^, it has been well established that the oncoproteins E6 and E7 are responsible for the onset and aggressiveness of the disease. Of these two proteins, E6 dysregulates the cell cycle, promotes hipper proliferation, induces malignant transformation, and facilitates migration and invasion of transformed cells in in vivo and in vitro studies^2^. Also, it has been proposed that the differences in the oncogenic potential of this virus is mediated by the genetic variation that occurs in the E6 gene, which alters the thermodynamics and structural stability on the 3D protein structure. However, the molecular insights into the differences in the interaction of this proteins with its targets remain unknown^3^.

E6 targets PDZ domains, and sometimes drives the proteasome-mediated degradation of these proteins that includes Dlg-1, Dlg-4, hScrib, MAGI-1, MAGI-2, MAGI-3, CAL, MUPP-1, PATJ, PTPN3, Tip1, and Tip2. The targeting of PDZ domain containing proteins has been shown to be a highly important activity in the process of carcinogenesis induced by HPV-16^25,36–45^. However, there is not plublish information about the differences in the interaction of any these proteins with variants of E6 of HPV-16, therefore were are interested in getting insights into the interaction of the five variants of E6 from HPV-16 with the cellular protein MAGI-1, Molecular Dynamics (MD) simulations and docking analyses were performed.

### 3D protein structures

**Figure 1:**
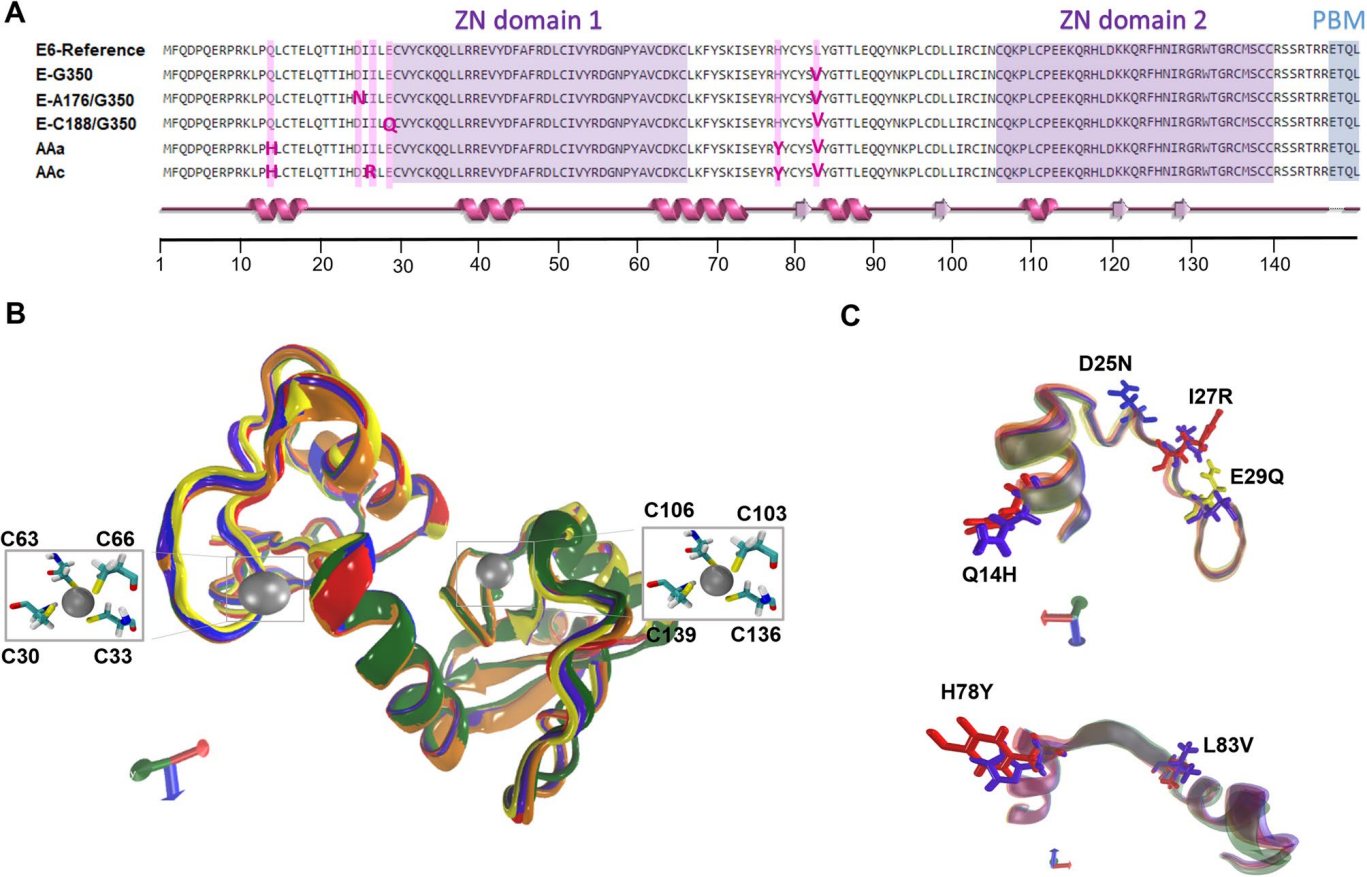
Alingment and super position of the 3D structures of the E6-reference and its variants. (**A**) Multiple alignment of the sequence of E6 and its variants. Zinc finger domain 1 and 2 are highlighted in purple, PBM in blue and mutations are highlighted in pink. A super position of the secondary structure of all six proteins is shown in pink below the alignment. (**B**) 3D structure of E6-reference: violet, AAa variant: orange, AAc variant: red, E-G350, green, E-C188/G350: yellow, E-A176/G350: blue. The silver spheres indicate zinc molecules and the licorice residues correspond to C30, C33, C63, C66, C103, C106, C136 and C139, which make up two zinc finger domains in the proteins 3D structures. The orientation of the proteins is indicated by the axes, X: red, Y: green; Z: blue. (**C**) Visualization of amino acid changes: Q14H, D25N, I27R, H78Y and L83V are in licorice.

Multiple alignments of the sequence of E6 and its variants were performed to evidence amino acidic changes between them. E6 mutations Q14H, D25N, I27R, E29Q and H78Y are found in a non-domain region adjacent to the zinc finger domain 1. While E6 mutations L83V and H78Y are located in an interdomain region between the two zinc finger domains (Fig. 1A).

The crystal structure of E6 PDB:4XR8 mutated to obtain the 3D structure of E6 reference, is composed of five alfa-helix and four beta-sheets. Also, it contains two zinc molecules forming two finger domains located at C30, C33, C63 C66, and C103, C106, C136, C139 residues (Fig. 1B). After in silico mutating E6-reference to obtained variants (E-G350, E-A176/G350, E-C188/G350, AAa and AAc) we compared them by an structural alignment using the VMD 1.9.3 RMSD tool (http://www.ks.uiuc.edu/Research/vmd/) (Fig. 1B). The RMSD values less than 2 Å represents accurate models. The RMSD values obtained for the variants were 0.51 Å for E-G350 (green), 0.66 Å for E-C188/G350 (yellow), 0.18 Å for E-A176/G350 (blue), 0.44 Å for AAa (red), and 0.69 Å for AAc (orange) (Fig. 1B). This mesuments were carried before the MD simulation to ensure that our mutating process did not damage their 3D strusture. All models were evaluated by Ramachandran plot showing that for all variants 91.9% of the amino acids falled within the favored region and 8.1% in the allowed region, which means a good stereochemistry for 100% of the residues (Fig. S1).

A close up of the mutated residues in the alignment shows that the side chains of mutants H14 and Y78 were exposed to the protein’s surface while the amino acids Q14 and H78 side chains of E6-reference were inverted (Fig. 1C). According to the program HOPE, which analyses the structural and functional effects of point mutations, H14 is bigger than Q14, bigger residues might lead to bumps on the 3D structure of the protein. H14 is among the observed mutations at this position in other homologous sequences. This sometimes suggests that the mutant is not damaging for the protein’s structure and function, on the other hand, the residue is located near a highly conserved position and can gain interactions with target proteins. The Y78 is bigger and more hydrophobic than the H78, this can result in loss of hydrogen bonds and disturbance of correct folding (Fig. 1C). The accessibility of the residues in the mutants could increase the number of interactions with the MAGI-1 models^46^.

Mutations E29Q and D25N remained the same size; therefore, not visible change in 3D structure was observed (Fig. 1C). A change in residue charge from negative to neutral can cause loss of interactions with other molecules or residues^46^. Mutant R27 is bigger than I27, this can be observed in the 3D structure comparison (Fig. 1C). Also, the change of a neutral to positive residue leads to the possibility of repulsion of ligands or other residues with the same charge. Moreover, the change of a hydrophobi residue to a neutral one will lead to the loss of hydrophobic interactions^46^. The mutation L83V found in all the variants can cause the proteins to lose interactions with other proteins because, V83 is a smaller residue than L83 (Fig. 1C), loss of interactions with cellular target proteins could lead to a diminution on the affinity of interactions for the variants^46^.

**Figure 2:**
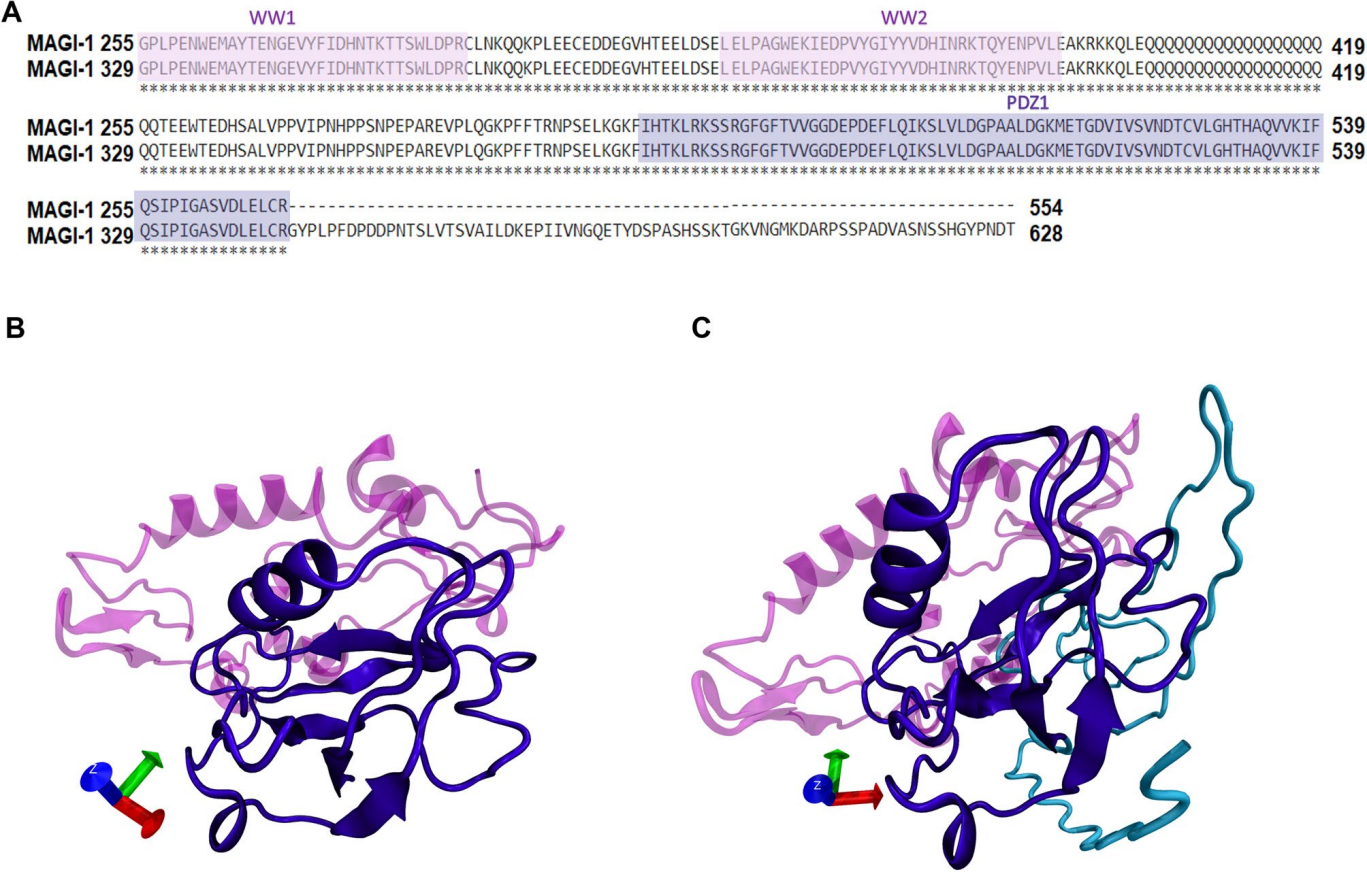
3D structure of MAGI-1 255 and MAGI-1-329 models. (**A**) Multiple alignment of the sequence of model MAGI-1 255 and MAGI-1 329 highlighting domains WW1 and WW2 in soft pink and domain PDZ1 in soft purple. (**B**) 3D model visualization of MAGI-1 255 (**C**) 3D model visualization of MAGI-1 329. The WW1 and the WW2 domains are shown in light pink, the PDZ1 domain is shown in violet, and a highly disordered region of 76 amino acids in cyan.

There are many crystal structures in the RCSB PDB server related to the crystalized MAGI-1; however, none of these files corresponds to the complete protein structure. Also, there are no reliable software for homology modelling such large proteins^32^. To overcome this drawback, we delimitated our models to domains that have been experimentally shown to interact with E6^14,25,39^. Our first model includes the WW1, WW2, PDZ1 domains and was denominated MAGI-1 255 and a second model where we added a highly disordered region of 76 amino acids adjacent to the PDZ1 domain denominated it MAGI-1 329. A sequence alignment of our final models is shown in (Fig. 2A). This additional region of 76 amino acids in our model 329 was added to compared the interaction of the variants with a bigger models of MAGI-1. According data publish by other groups, the interaction of E6 with MAGI-1 occurs mainly with the PDZ-1 (H472-R554) domain, but different affinity patterns were observed between adjacent regions closed to the PDZ-1 domaninand E6^28–30^. Also, computational approaches have highlighted the role of distal regions of the E6 proteins to form a dynamics networks within PDZ domains, and a number of studies have characterized these changes in dynamics using NMR, thus providing experimental evidence for this interaction^47–49^. Taking into account all the avalible information, we included WW1, WW2 and their interdomains to our models of MAGI-1. Besides these domains we also, added a highly disordered region of 76 amino acids (G555–T628) to model MAGI-1 329 shown in cyan, this region was reported to have key role in the interaction of this proteins^29^. With this we incremented the coverage of MAGI-1 in this model and predicted that this region increments the affinity of E6 and its variants to MAGI-1.

The 3D homology models of MAGI-1 255 (amino acid 300–554) and of MAGI-1 329 (amino acid 300–628) were obtained using the crystal structure from human MAGI-1 PDZ1 (PDB ID:2KPK)^28^ as a template on the I-TASSER server^50^ (Fig. 2B,C). The best models were chosen according to the criteria of good alignment with the template measured by C-Score, TM score, and RMSD values. Model MAGI-1 255 shown in Fig. 2B consists of six alfa-helix, one 310 helix, eleven beta-sheets and the rest of the residues appeared lightly twisted in random coils, which are 15.3%, 2.4%, 13.7% and 68.6% respectively of the protein structure. The two WW domains and the interdomain regions from amino acid G300 to amino acid I471 are shown in magenta, and the PDZ1 domain from amino acid H472 to amino acid R554 is shown in purple (Fig. 2B). Model MAGI-1 329 consists of seven alfa-helixes, twelve beta-sheets, and the rest of the residues appeared in loops and coils, which are 15%, 14.1%, and 70.9%, respectively the protein structure. For this model, the 3D structure from amino acid G300 to R554 are in purple, and the extra region of 76 (G555–T628) amino acids are in cyan (Fig. 2C).

Ramachandran plot for model MAGI-1 255 and MAGI-1 329 exhibited 92.8% and 91.4% respectively of residues in most favored regions and 7.2% and 8.6% respectively residues are in disallowed regions, which shows a good stereochemistry for more than 90% of the residues, this makes our models acceptable for more refinement with MD simulation (Fig. S2).

### Molecular dynamics simulation analysis

**Figure 3:**
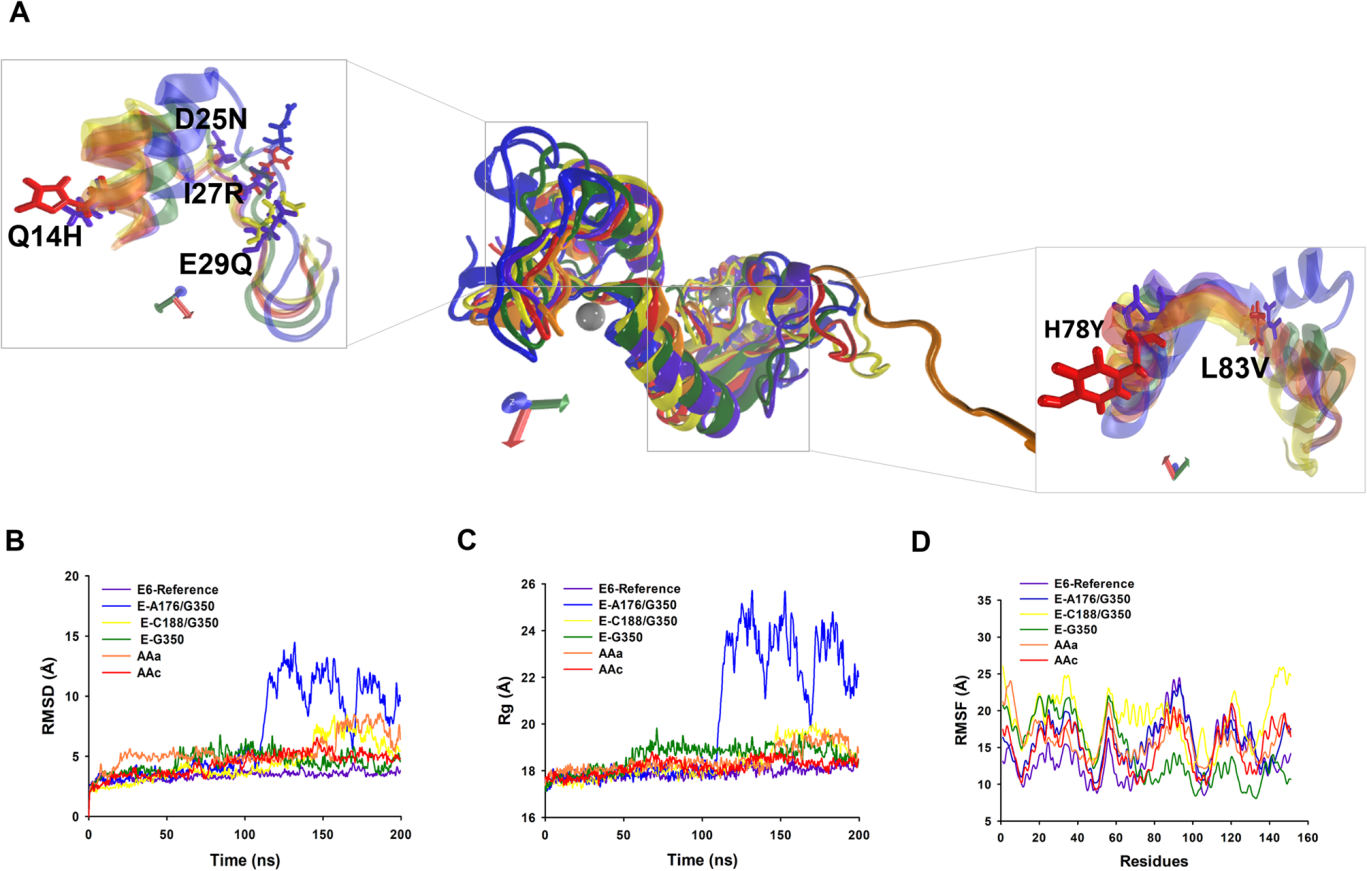
3D structures of the E6-reference and its variants and conformational stability during 200 ns MD simulation. (**A**) Super position of average 3D structures of HPV-16 and its variants. Zoom visualization of amino acid changes: Q14H, D25N, I27R, E29Q, H78Y and L83V. E6-reference: violet, AAa variant: orange, AAc variant: red, E-G350, green, E-C188/G350: yellow, E-A176/G350: blue. The silver spheres indicate zinc molecules. The orientation of the proteins is indicated by the axes, X: red, Y: green; Z: blue. (**B**) RMSD. (**C**) Radius of gyration. (**D**) RMSF.

To examine the change in the protein dynamics and stability, the 3D models of HPV-16 E6 and its variants, as well as MAGI-1 255 and MAGI-1 329 were refined by MD simulation for 200 ns. Trajectories were analyzed by calculating the root mean square deviation of atomic positions (RMSD), root mean square fluctuation (RMSF), the radius of gyration (Rg), the dPCA analysis and dPCA based clustering (Fig. 3).

After 200 ns of the MD simulation and using a snapshot of the most populated cluster of E6 and variants, a structural alignment was done (Fig. 3A). The carboxyl terminus of the E-C188/G350, AAa and AAc proteins showed a greater difference compared to E6-reference, while the other variants 3D structures remained very similar compare to E6-reference (Fig. 3A).

The RMSD calculation of the E6-reference (purple) during the 200 ns of MD simulation, reached equilibrium at 20 ns of trajectory, while the non-European variants AAa (orange) and AAc (red) were equilibrated at 80 ns, after 150 ns AAa variant loses its equilibrium and recovers it at the end of the DM simulation, probably due to its mutations and its context (Fig. 3B). Something similar happens to the variant E-C188/G350 (yellow), which reaches equilibrium at 60 ns, and its equilibrium is disturbed from the 150 ns to the 180 ns. This behaviour is attributed to mutations in the E29A and L83V positions that directly affect the structure of the protein, causing disturbs in it’s stability (Fig. 3B). Concerning E-A176/G350 (blue), the equilibrium was reached at the first 20 ns, but a greater disturbance episode it’s observed from 100 ns to the end of the trajectory it is also thought that the nature of mutation D25N in the proteins may contribute to this behavior. It was also observed that for E6-reference, E-G350 (green), and E6-AAc, the RMSD values during the simulation ranged from 2 to 5 Å (Fig. 3B). While variants AAa, E-A176/G350 and E-C188/G350 were characterized by higher continuous RMSD values from the 140 ns to the end of the MD simulation (Fig. 3B). The RMSD values of simulated proteins indicated their stability and particular behavior and provided a suitable basis for further analysis.

The Rg presents different grades of compactness during the simulation evidencing a less compactable grade at the end of the trajectory, mainly in the variants E-A176/G350, E-C188/G350 and AAa (Fig. 3C in yellow and orange). Meanwhile, E6-reference and variants E-G350 and AAc maintain compactness during the simulation (Fig. 3C, green and red). This also confirms that point mutations caused structural destabilizing effects leading to the loss of protein compactness in the E-A176/G350, E-C188/G350 and AAa variants. Since distance deviations from the starting structure may not necessarily reflect mobility of structural elements, RMSF was used to obtain information on flexibility. According to the data graph in Fig. 3D, there are six maximum fluctuations peaks areas shared by E6-reference and variants: One at M1 to P5, for E6-reference the fluctuation peak was 16 Å, and for the variants, the highest peak corresponds to E-C188/G350 with 27 Å, the rest of the variants fluctuate from 14 to 24 Å of distance being E-G350 the lowest peak, this region is composed by coils and turns with non-secondary structure (Fig. 3D). The second region at L28–L50 composed of loops and an alpha helix: reached 15 Å for E6-reference and it wasthe highest fluctuation peak. Fluctuation for variants ranges from 20 to 25 Å; clearly the variants had greatest fluctuation in these residues, where E-C188/G350 had the greates fluctuation peaks (Fig. 3D). This phenomenon is interesting and it’s attributed to mutation E29Q exclusive of the E-C188/G350 variant, while the behavior of the other variants is exclusive of their own structural changes caused by their shared and exclusive mutations. The third peak at C51–L65, in a coil and two beta-sheets, the variants reached 20–23 Å of fluctuation peaks, while E6-reference’s fluctuation was only 16 Å (Fig. 3D). The fourth region of fluctuation at C80–L110, was composed of loops, one alpha helix and two beta-sheets and reaches 25 Å for variant E-A176/G350 and E6-reference. For variants AAa and AAc the fluctuation distance was 20 Å. While the peak for variant E-G350 was only 14 Å, evidently less flexiblethan the other variants and E6-reference (Fig. 3D). The fifth fluctuation peak at C111–C140, in two beta sheets, coils and an one alpha helix, for variants AAa, AAc, E-C188/G350, E-A176/G350 had a fluctuation distance of 20 Å. E6-reference, also, reached a distance of 20 Å. On the other hand, the distance of E-G350 reached 15 Å, and it tends to decrease for the rest of the amino acids at the carboxyl terminus (Fig. 3D). Finally the residue-based RMSF of the backbone for the E6-reference displayed less flexible residues than the variants (E-G350, E-C188/G350, E-A176/G350, E6-AAa, and E6-AAc), at the carboxyl terminus (145–151) composed mainly by loops (Fig. 3D). Interestingly, this region includes the PBM (ETQV) motif of E6, which is important for this oncoprotein interaction with MAGI-1. Since there is a higher fluctuation in variants compares to E6-reference, it can be deduced that mutations change the structural flexibility of the 3D protein structure.

Ramachandran analysis after the MD simulation of these structures shows that more than 98% of the amino acids of the proteins during the simulation remain in the highly favored regions, which means that the protein’s conformation are well refined and have native conformations (Fig. S3).

**Figure 4:**
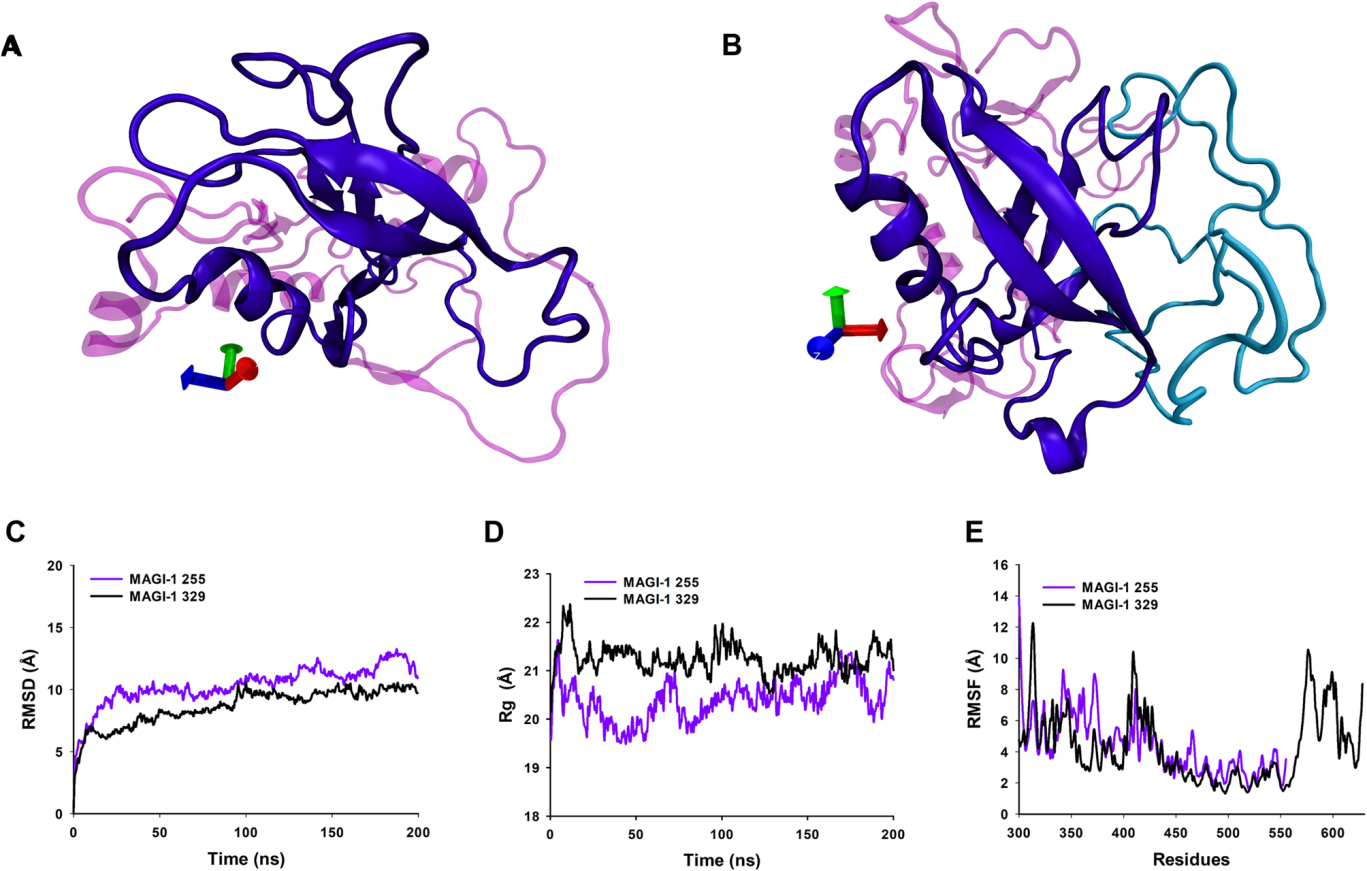
3D structures of MAGI-1 255 and MAGI-1 329 and conformational stability during 200 ns MDs. (**A**) Visualization of MAGI-1 255. (**B**) Visualization of MAGI-1 329. The WW1 and the WW2 domains are shown in light pink, the PDZ1 domain is shown in violet, and a highly disordered region of 76 amino acids in cyan. (**C**) RMSD. (**D**) Radius of gyration. (**E**) RMSF.

For our two MAGI-1 models we showed a snapshot of the most populated cluster from the dPCA clustering analysis in (Fig. 4A,B). The PDZ1 domain of our two models have little changes in its 3D structure, but overall it keeps its main 3D structure (Fig. 4A,B).

The RMSD of MAGI-1 255 (purple) and MAGI-1 329 (black) models during the 200 ns trajectory showed that both models reached equilibrium before the 100 ns of the simulation and continue stable for the rest of the trajectory. Moreover, MAGI-1 255’s RMSD value after equilibrium ranges from 10 to 13 Å and MAGI-1 329 model’s RMSD values range from 9 to 10 Å, which means our models are reliable for further investigation (Fig. 4C).

The Rg show that both models maintained a compacted structure throughout the trajectory of MD simulation (Fig. 4D). We explored the flexibility of the models by measuring Cα. The RMSF values of the models through trajectory, mainly 3 regions of MAGI-1 255-model showed more flexible areas, those regions correspond to amino acids G300 to A309 of the WW1 domain, G349 to D379 belong to the WW2 domain and Q399 to H429 that correspond to an interdomain region between the WW2 and PDZ1 domains of MAGI-1 (Fig. 4E). MAGI-1 329 model showed three regions with more flexibility which include amino acids E304 to I319 of the WW1 domain, Q399 to V433 corresponding to an inter domain between WW2 and PDZ1 domains and N566 to T628, this region was the most flexible of the two models. Interestingly, this region corresponds to a highly disordered region of the whole protein (Fig. 4E)^29^. The Ramachandran analysis shows the refinement of more than 80% of the model’s residues (Fig. S4).

### Dihedral principal component analysis

**Figure 5:**
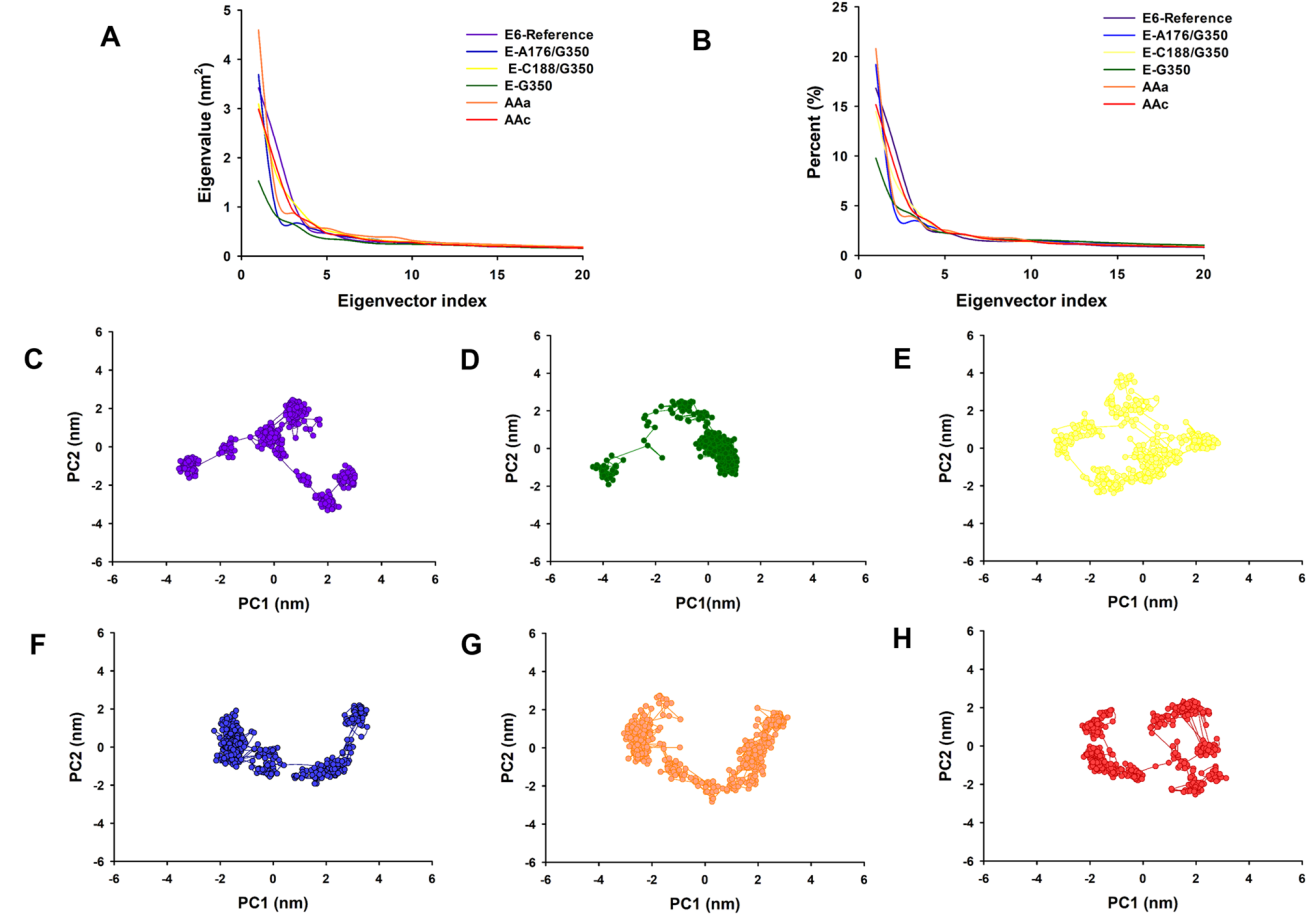
Principal component analysis (PCA) of E6-reference and its variants from HPV-16. (**A**) The eigenvalues plotted against the corresponding eigenvector indices obtained from the Cα covariance matrix constructed from the 200 ns MD trajectory. E6-reference: violet, AAa variant: orange, AAc C variant: red, E-G350, green, E-C188/G350: yellow, E-A176/G350: blue. (**B**) Percentage of each eigenvector vs. eigenvalues. E6-reference: violet, AAa variant: orange, AAc C variant: red, E-G350, green, E-C188/G350: yellow, E-A176/G350: blue. Projection of the motion of the structures of the backbone atoms (PC1 vs PC2) (**C**) E6-reference. (**D**) E-G350. (**E**) E-C188/G350 (**F**) E-A176/G350. (**G**) AAa and (**H**) AAc.

dPCA was used to obtain a broader view of dynamic properties with respect to MD simulation results of E6-reference and its variants, MAGI-1 255 and MAGI-1 329. The covariance matrix for the first 20 eigenvectors of E6-reference was 11.40 nm^2^ and 10.09 nm^2^, 11.14 nm^2^, 7.12 nm^2^, 12.23 nm^2^ and 10.60 nm^2^ for the variants E-G350, E-A176/G350, E-C188/G350, AAa and AAc, respectively (Fig. 5A). Moreover, the dPCA analysis showed that the first 20 eigenvectors captured 45–57% of the total protein motions (56.7, 45.5, 52.5, 51.1, 55.3 and 53.8%) for E6-reference, E-G350, E-A176/G350, E-C188/G350, AAa and AAc respectively (Fig. 5B). Whereas the projections of the first two principal components (PC1 vs PC2) contributed to 15–28% of the collective motions (28.22, 15.11, 24.20, 22.40, 26.66 and 24.59%) for E6-reference E-G350, E-A176/G350, E-C188/G350, AAa and AAc respectively (Fig. 5B). There are changes in the motions of the atoms of the variants E-A176/G350, AAa and AAc compared to E6-reference. Moreover a considerable change in the motion of the atoms of G350 and E-C188/G350 compared to E6-reference, which suggests that the properties of the movements described by the first PCs were different in the six protein systems (Fig. 5A,B).

The projection of the first two eigenvectors (PC2 vs. PC1) for E6-reference, E-G350, E-A176/G350, E-C188/G350, AAa and AAc (Fig. 5C–H), shows that E6-reference system (Fig. 5C) present different mobility behavior compared to the variants systems. The variants E-G350, E-C188/G350 and AAc have more restricted motions, making them the more stable of the six protein systems (Fig. 5D,E,H). The variants E-A176/G350 and AAa were expanded in their conformational space due to their flexibility (Fig. 5F,G). This points out that the punctual mutations of residues affect conformation and motion.

**Figure 6:**
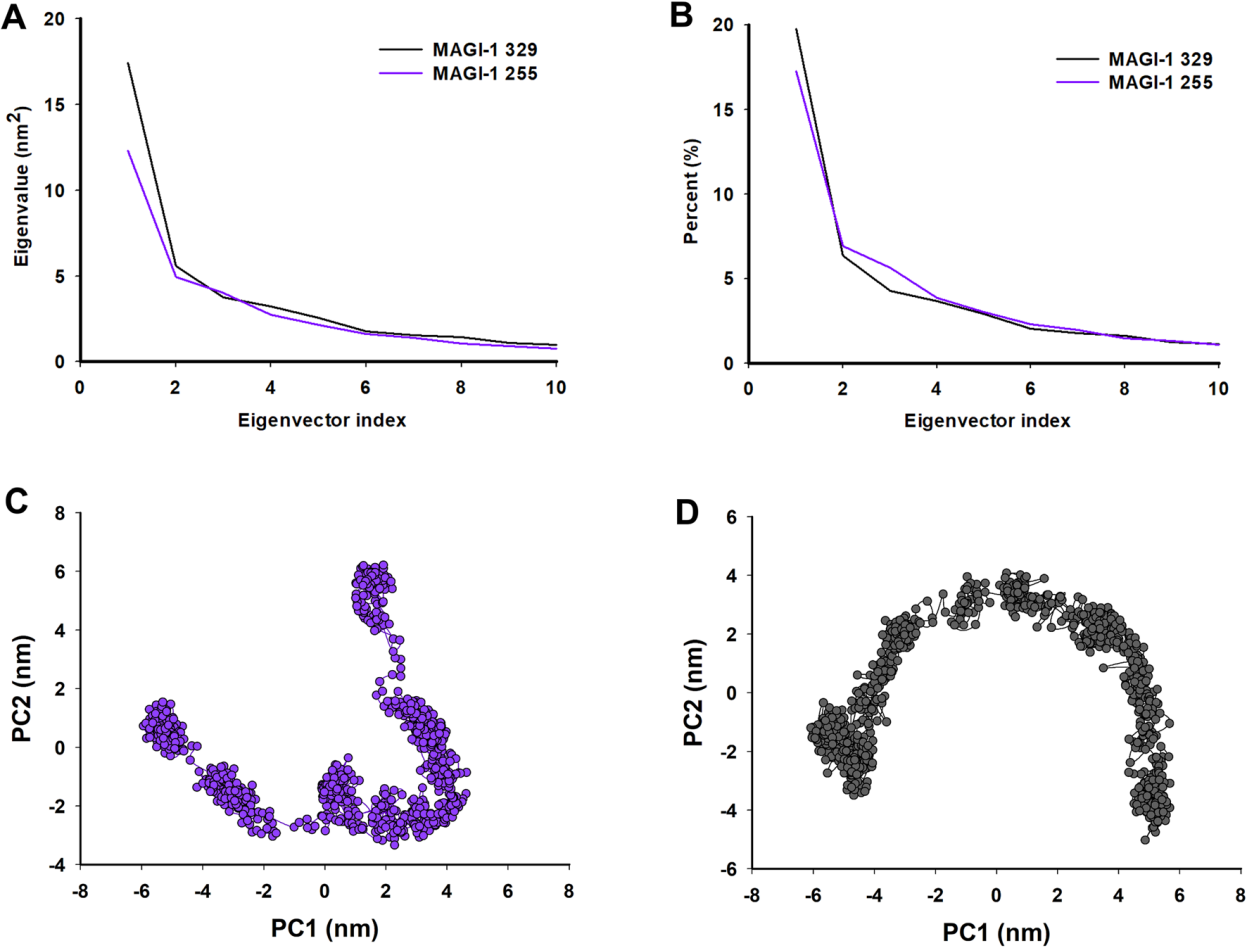
Principal component analysis (PCA) of MAGI-1 255 and MAGI-1 329. (**A**) First ten eigenvalues plotted against the corresponding eigenvector indices obtained from the Cα covariance matrix constructed from the 200 ns MD trajectory. MAGI-1 255 purple and MAGI-1 329 black. (**B**) Percentage of each eigenvector vs. eigenvalues. 2D Projection of Principal Component Analysis. Projection of the motion of the protein in phase space along the first two principal components. (**C**) MAGI-1 255 and (**D**) MAGI-1 329.

With respect to MAGI-1 255 and MAGI-1 329 the matrix value obtained for the for the models were of 17.4 nm^2^ and 12.3 nm^2^ and the dPCA analysis showed that the first 10 eigenvectors captured 22.7 and 34.7% of the proteins total motions (Fig. 6A,B). The projection of the first two eigenvectors (PC2 vs. PC1) for MAGI-1 255 and MAGI-1 329 shows differences in their mobility behaviour (Fig. 6C,D). These results showed a considerable change in the motion of the atoms of MAGI-1 255 and MAGI-1 329, which means that the missing 76 amino acids of model 255 restricts its motions, making it more stable. These results support Ramírez et al. observations about the contribution of the highly disordered 76 amino acid region adjacent to the PDZ-1 domain of MAGI-1 to its behavior^29^.

### Protein–protein docking

Mutations in proteins can affect protein structure and stability, consequently, these mutations alter the kinetics and thermodynamics of protein–protein interactions (PPI)^51^. Using ClusPro blind base docking method, a representative protein structure of the most populated cluster obtained from the dPCA clustering analysis of the MD simulation refined proteins (E6-reference, E-G350, E-A176/G350, E-C188/G350, AAa and AAc) were docked against the representative structure of MAGI-1 255 and MAGI-1 329, also, obtained from dPCA clustering analysis. Docking resulted in 1000 protein conformations of complexes. The top ten docked complexes from each problem were analyzed for the lowest energy and residues binding between the two proteins. The best complexes were selected base on a greater number of cluster members and the lowest energy according to ClusPro guidelines^43^.

The global free binding energy of the E6-reference against 255 complexes and MAGI-1 329 were calculated as − 48.14 and − 51.90 kcal/mol respectively, using FiberDock^52^. These energies were bigger than the energies obtained from the complexes between the E6 variants and the MAGI-1 models; this means that there is a greater affinity between MAGI-1 models and E6 variants compare to E6-reference (Table 1). However, the variants that presented the lowest binding energy with the MAGI-1 255 model were E-A176/G350, AAa and E-C188/G350 (− 191.34, − 138.07 and − 130.89, respectively). Meanwhile variants AAc, E-G350, and E-A176/G350 and against MAGI-1 329 showed the lowest energy values (− 166.97, − 152.50 and − 148.86, respectively). We interpreted this as a gain of interaction affinity between these proteins. In conclusion, the lowest energy docking values was between MAGI-1 255 and variant E-A176/G350 (Table 1). In addition, there was an increment in the number of hydrogen bond in the complexes formed by the variants and both models of MAGI-1 compared to the E6-reference. However the number of salt bridges interactions only increased in the complexes G-350, E-C188/G350 and E-A176/G350 with MAGI-1 255 compared to E6-reference. Concerning the complexes between E6-reference and its variants with MAGI-1 329 only variants AAa and E-C188/G350 increased their salt bridges interactions (Table S1). Therefore, we concluded that the variants gain affinity to our two models of MAGI-1.

**Table 1:** Docking binding affinity of E6 HPV-16 and its variants.

Complexes	No. cluster members’	Binding energy (kcal/mol)
E6R/MAGI-1 255	83	− 48.14
E-G350/MAGI-1 255	126	− 111.14
E-C188/G350/MAGI-1 255	111	− 130.89
E-A176/G350/MAGI-1 255	74	− 191.34
AAa/MAGI-1 255	111	− 138.07
AAc/MAGI-1 255	204	− 120.23
E6R/MAGI-1 329	118	− 54.90
E-G350/MAGI-1 329	88	− 152.50
E-C188/G350/MAGI-1 329	82	− 121.63
E-A176/G350/MAGI-1 329	91	− 148.86
AAa/MAGI-1 329	83	− 148.17
AAc/MAGI-1 329	88	− 166.97

**Figure 7:**
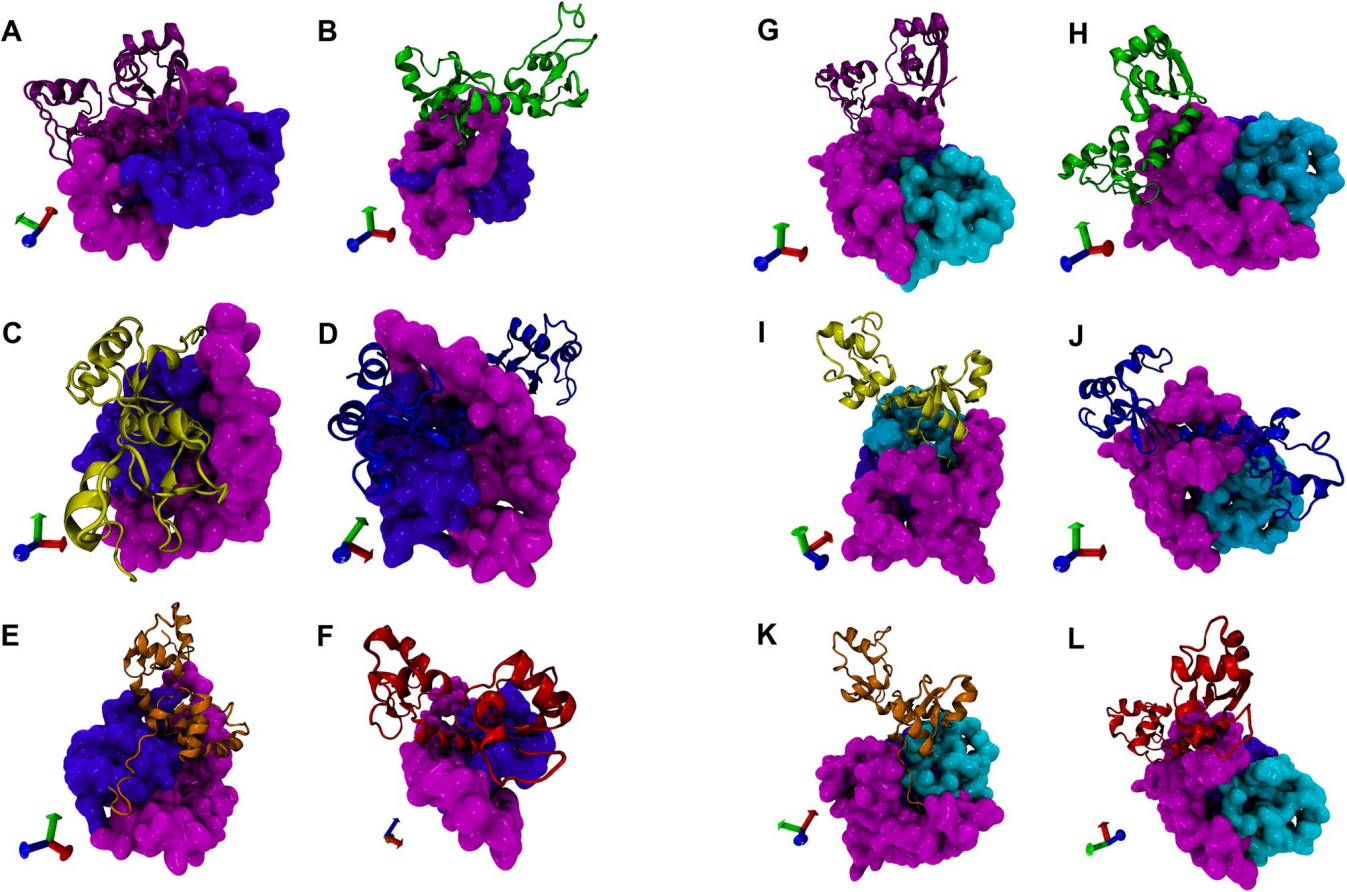
Protein–protein docking of E6, its variants with MAGI-1 255 and MAGI-1 329. Protein–protein docking analysis shows the probable interaction of E6-reference (purple), E-G350 (green), C188/G350 (yellow), E-A176/G350 (blue), AAa (orange) and AAc (red) with MAGI-1 255 (**A**–**F**). Docking between MAGI-1 329 and E6-references and its variants (**G**–**L**) E6-reference (purple), E-G350 (green), C188/G350 (yellow), E-A176/G350 (blue), AAa (orange) and AAc (red). MAGI-1 255 and MAGI-1 329 are represented in quicksurf in color magenta. The protein–protein docking was performed using the ClusPro 2.0 web server.

The protein–protein interfaces of the complexes were analyze using PDBsum generate and are shown in Fig. 7A–L^53^. The top docked complex of each variant against MAGI-1 255 and 329 were subjected to PDBsum to identify the interacting residues. Comparative analysis between the twelve complexes from docking interfaces of E6-reference and its variants identified a list of different amino acids that were shown to be responsible for the interaction with the MAGI-255 and MAGI-1 329 (Table S2).

For the complex E6-reference and MAGI-1 255 (Fig. 7A), the interaction occurs mainly through amino acids of the WW2 domain (Y74, V79, D80, W66, G65, A64, Y78, I76) and from the PDZ1 domain (A234, H231 and G230) with twenty-four amino acids of E6-reference that included: Y81, R77 and Y76 which are adjacent to H78 a highly mutated amino acid in E6 (Table S2). There are nine hydrogen bonds and three salt bridges (Table S1). On the other hand, the interaction of E6-reference with MAGI-1 329 was through amino acids corresponding to WW2 domain and adjacent non-domain regions (Table S2). MAGI-1 329 amino acids C34 to F171 were responsible for most of the interactions with twenty-three amino acids of E6-reference that included: R147, R131, H78, R77, Y76, T32 and V31 (Fig. 7G). Some of the E6-reference interacting residues are located closed to the mutation sites, and its interacting residues are different compared to the variants.

The differences between the interaction of E6-reference with our two models from MAGI-1 is remarkable, the increase in protein’s coverage results in a gain of interacting residues, an increase of hydrogen bonds, Salt bridges (Table S1).

For complexes E-G350/MAGI-1 255 (Fig. 7B), E-G350/MAGI-1 329 (Fig. 7H), E-A176/350/MAGI-1 255 (Fig. 7D), E-A176/350/MAGI-1 329 (Fig. 7J), AAc/MAGI-1 255 (Fig. 7E) and AAc/MAGI-1 329 (Fig. 7K) detailed information about the amino acids involved in the interaction and the type of bonds can be found in Tables S1 and S2.

We observed that in complex E-C188/G350/MAGI-1 255 (Fig. 7C) the interactions of the PBM motif (E148, T149, Q150, and 151L) were lost, but, interestedly the interacting residues from complex E-C188/G350/MAGI-1 329 (Fig. 7I) included all the amino acids of the PBM motif of E6 (E148, T149, Q150, and 151L) these residues interact mainly with amino acids from the WW1 (G1-R33) domain and amino acids from a highly disordered region of MAGI-1 (G256-T329 in our model) which agrees with the experimental evidence publish by Ramirez et al.^29^ (Table S2).

It is important to point out that H78 is mutated to T78 in the Asian American variants this mutation causes the lost of interaction between this residue and our models of MAGI-1 only for AAa variant, meanwhile, AAc variant does not exhibit this behaviour. We observed that the interaction of AAa variant with MAGI-1 255 was not conducted throught its PBM motif (Fig. 7F). Meanwhile, the interaction of this variant with MAGI-1 329 included all the residues from the PBM motif (R147, E148, T149, Q150 and L151) (Fig. 7L).

Regarding the missing interaction of the PBM motif with MAGI-1 255 and 329, we believe that the residues could be oriented in a way that avoids the interaction or may be block by adjacent residues. According to the online server HOPE, this could be attributed to changes in size and in charge from the mutations of each variant^46^.

It is important to understand the changes in PPIs caused by these mutations may alters the affinity and stability of the interaction of E6 with proteins important for tissue homeostasis. The increase in affinity and stability of the interaction of E6 with MAGI-1 result in an increase in the degradation of MAGI-1 and as a consequence in the loss of stability of important cell complexes that maintain cell–cell adherence at the adherents junctions.

## Materials and methods

### 3D protein structures

Multiple alignments of the sequences (Accession number P03126) were performed using CLUSTAL X 1.81^54^. The secondary structure of the E6 protein and its variants were predicted using PSIPRED server^55^. The crystal structure of HPVt-16 E6 protein was obtained from the Protein Data Bank (RCSB PDB)^56^, with the identification number: 4XR8, chain H^21^. The E6 structure on this PDB contains 151 residues with four-point mutations in S80C, S97C, S111C, and S140, which were reverted to obtain the E6 reference in the PyMOL Molecular Graphics System, Version 2.0 Schrödinger, LLC^57^. After that, all the mutations were carried out in the E6 reference to obtained all the variants of HPV-16. The mutations were done as indicated next, to obtained E-G350: L83V; E-C188/G350: E29Q and L83V; E-A176/G350: D25N and L83V; AAa: Q14H, H78Y, and L83V; for AAc: Q14H, I27R, H78Y, and L83V. The obtained proteins were structurally aligned and visualized using VMD 1.9.3^58^.

To obtained the 3D structure of MAGI-1, a total of 255 and 329 amino acids from the amino acid terminal region of the protein sequence (300–554 and 300–628) were retrieved from the UniProtKB database^59^, (accession number Q96QZ7) and submitted to I-TASSER server as two separate jobs^50^. First, the 3D structure with 225 residues, which comprises WW1, WW2 and the PDZ1 domains of MAGI-1 was obtained by homology modelling using the I-TASSER server (https://zhanggroup.org/I-TASSER/), as a template, we selected PDB file: 2KPK^28^, which corresponds to the PDZ-1 domain of the MAGI-1. Furthermore, a 3D structure of 329 amino acids of MAGI-1, which includes the WW1, WW2, PDZ-1 and a 76 amino acidic disordered region of this protein, was obtained by homology modelling on the I-TASSER server using the same template PDB. All the 3D predicted structures were evaluated using the Rampage webserver to obtain the Ramachandran plots (http://mordred.bioc.cam.ac.uk/~rapper/rampage.php).

### Molecular dynamics simulation

Parameters for the two Zn^2+^ ions and eight cysteine-ligand coordination of E6 were kindly provided by Justin Lemkul from the Virginia Polytechnic Institute and State University, these parameters included a CYSD patch for the deprotonation of the eight zinc-bound cysteines and a ZN_C patch to covalently link cysteines to the zinc ions. The correct coordination of the deprotonated cysteines and the ion zinc using these patches has been demonstrated in previous studies^60^, the CHARMM 36 force field was employed for the application of the patches using CHARMM software (http://charmm.chemistry.harvard.edu/charmm_lite.php)^61,62^. Afterwards, a 200 ns of MD simultion of E6 and its variants were performed using the NAMD 2.8 (http://www.ks.uiuc.edu/Research/namd/) software package^63^ with CHARMM36 and CHARMM22 force fields^62^. For MAGI-1 models we used CHARMM 27 topology and parameter files for proteins. Each system was placed in a cubic box of TIP3P water with a minimum distance of 10 Å between the solute atoms and the edge of the box^64^. To neutralize the systems, we added 7568 water molecules, 21 Na+ and 27 Cl− to the E6-reference. To variant E-G350, we added 7420 water molecules, 21 Na+, and 27 Cl−, to E-C188/G350 variant, 7484 water molecules, 21 Na+, and 28 Cl− were added, to E-A176/G350 variant, 7660 water molecules, 22 Na+ and 29 Cl− were added, to AAa variant 7566 water molecules, 21 Na+ and 27 Cl− were added and to AAc variant, 7554 water molecules, 21 Na+ and 28 Cl− were added. For MAGI-1 255 we added 10,932 water molecules and 18 Sodium, and for MAGI-1 329, 11,456 water molecules and 23 Na were added. Each system was neutralized to 0.15 mol/L of NaCl and submitted to minimization energy for 10,000 steps of steepest descent minimization followed by equilibration for 1 ns under constant temperature 310 K and pressure 1 atm (NPT) ensemble with protein atoms restraints^65,66^. MD simulation were run for 200 ns, considering all proteins as soluble.

### Trajectory and dPCA analysis

The carma software (https://utopia.duth.gr/~glykos/Qs.html)^67^, was used to calculate the root mean square deviation (RMSD) calculates the average deviation in the atomic stability throughout MD simulation, radius of gyration (Rg) measures the compactness and expansion of the molecules, and the root means square fluctuation (RMSF) a parameter to explored the flexibility of the protein through MD simulation, as well as the Principal component analysis (PCA) and dPCA based clustering analysis employing the last 50 ns of the trajectory. dPCA is a standard tool in statistical mechanics used in order to determine the correlated motions of the residues to a set of linearly uncorrelated variables called principal components, and it allows to obtain the large scale collective motions of the atoms on the simulations, which frequently correlates with the proteins biological function and structural properties^68^. Finally, we obtained the PDB files from the most populated cluster analysis and performed a protein–protein docking. Molecular graphics were performed in Sigma plot 12.0. VMD 1.9.3 was used to visualize all the 3D proteins^58^.

### Protein–protein docking

The protein–protein dockings were carried in Cluspro server (https://cluspro.bu.edu/login.php)^69,70^, the program has been consistently rated among the best global docking methodologies in the CAPRI challenge (Critical Assessment of Predicted Interactions)^69^. For the docking studies, refined models for most populated cluster from E6-reference or its variants were docked within the MAGI-1 (235 and 329) homology models, where MAGI-1 models were the receptors and E6, and its variants were used as a ligand. The conformers with the highest cluster members and the lowest energy calculated in FireDock were taken for analysis on the PDBSum server^52,53^. All docking complexes were visualized by VMD 1.9.3 software^58^.

## Conclusions

We proposed an in-silico approach to evidence the differences in the interaction of E6 and five of its natural variants with two models, cellular protein MAGI-1. According to our results variants, AAa and E-C188/G350 showed less RMSD values, less compactness, a gain of fluctuation regions that are correlated to the increment of active sites. We attribute this behavior to specific mutations of proteins, and these mutations cause physicochemical changes that affect the behavior of proteins. Very marked dynamic changes are observed, particularly at the amino and carboxyl termini of proteins, where there is a gain in flexibility in the variants compared to E6-reference. Also, according to the dPCA results a dramatic change of motions behaviour for mutants compared to E6-reference. These differences in structure and mobility incremented the affinity of variants E-C188/G350 and AAa for our models of MAGI-1. E-C188/G350 increases its affinity for our models by three times, increasing the binding bonds by 50%. A similar pattern is observed among all the variants compared to E6-reference. Our results suggest that the physicochemical changes that gave rise to thermodynamic changes of the variants and an increase the affinity for our MAGI-1 models. Here, we were able to represent the possible changes in the physicochemical properties of E6 proteins and the repercussion in the interaction affinity with MAGI-1. An experimental validation will be necessary to evaluate the degradation profile of the MAGI-1 protein mediated by E6-reference and its variants.

## Supplementary Information

Supplementary Information.
