# Supplementary material for: Molecular insights into the interaction of HPV-16 E6 variants against MAGI-1 PDZ1 domain
Source: Sci Rep. 2022 Feb 3;12:1898. doi: 10.1038/s41598-022-05995-1 (PMC8814009; doi:10.1038/s41598-022-05995-1)
Supplement: Supplementary file 1 — Supplementary Information. [file 41598_2022_5995_MOESM1_ESM.pdf]

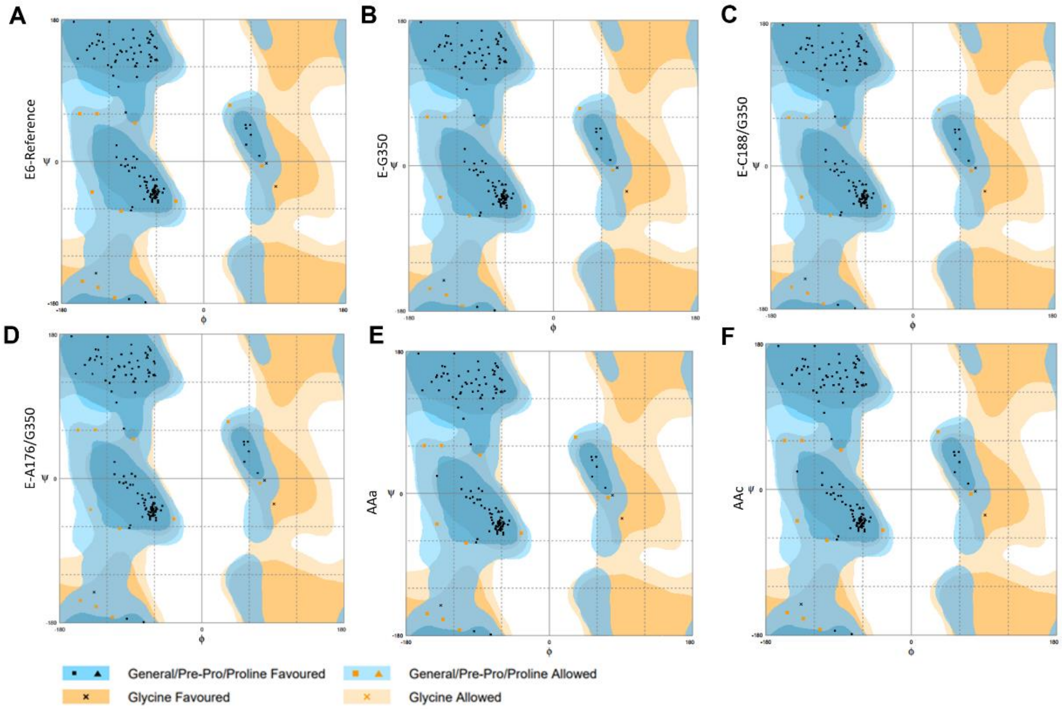

S1 Figure. Ramachandran plot analysis of E6 and its variants from HPV-16. (A) E6-reference. (B) E-G350. (C) E-C188/G350. (D) E-A176/G350. (E) E6-AAa. (F) E6-AAc.

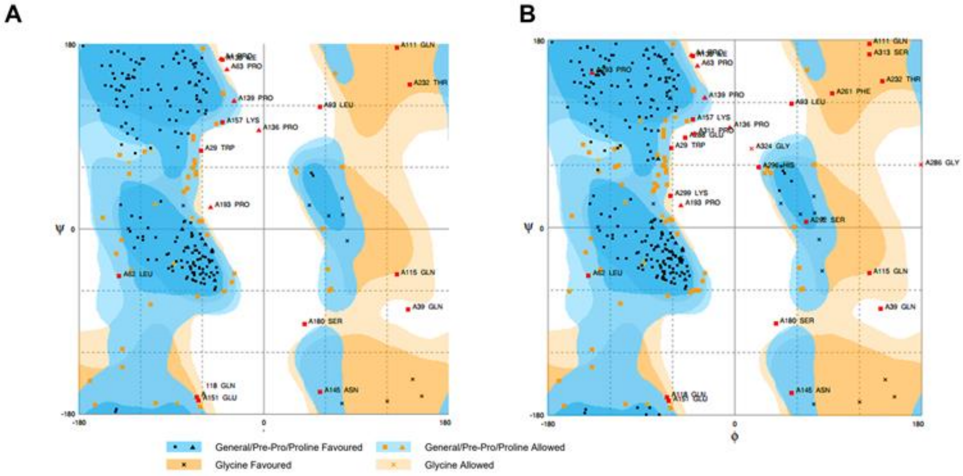

S2 Figure. Ramachandran plot of MAGI-I. (A) Ramachandran plot of MAGI-1 255 model from I-TASSER. (B) Ramchandran plot of I-TASSER model of MAGI-1 329.

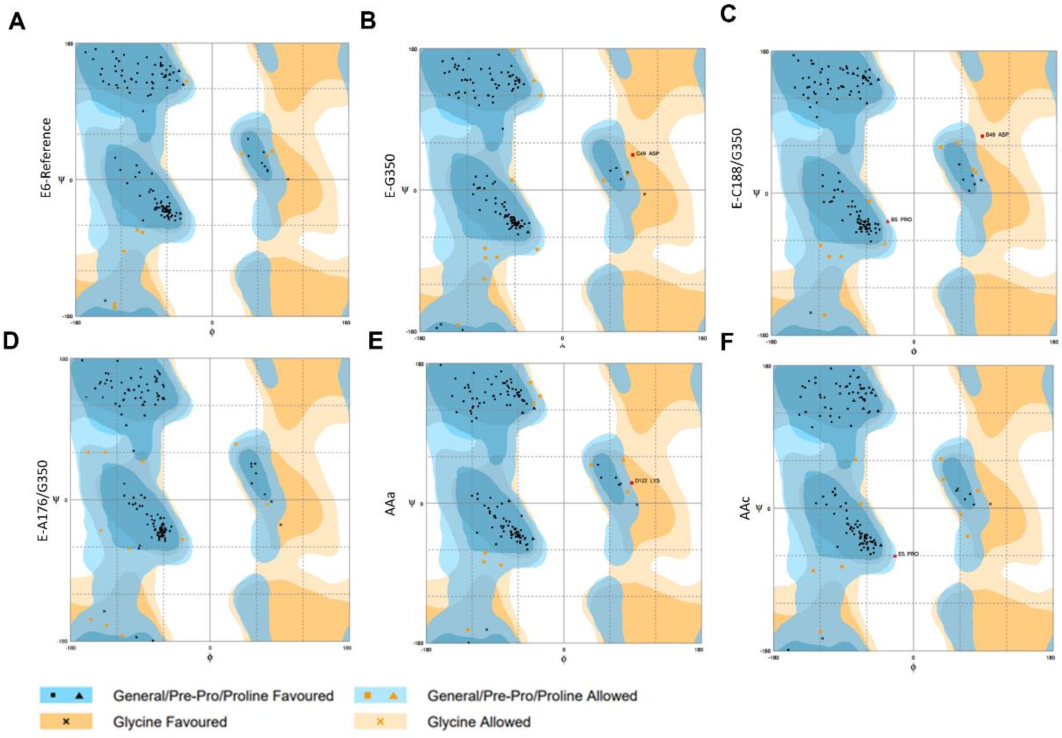

S3 Figure. Ramachandran plot analysis of E6-reference and its variants after MD simulation. (A) E6-reference (B) E-G350. (C) E-C188/G350. (D) E-A176/G350 (E) AAa (F) AAc.

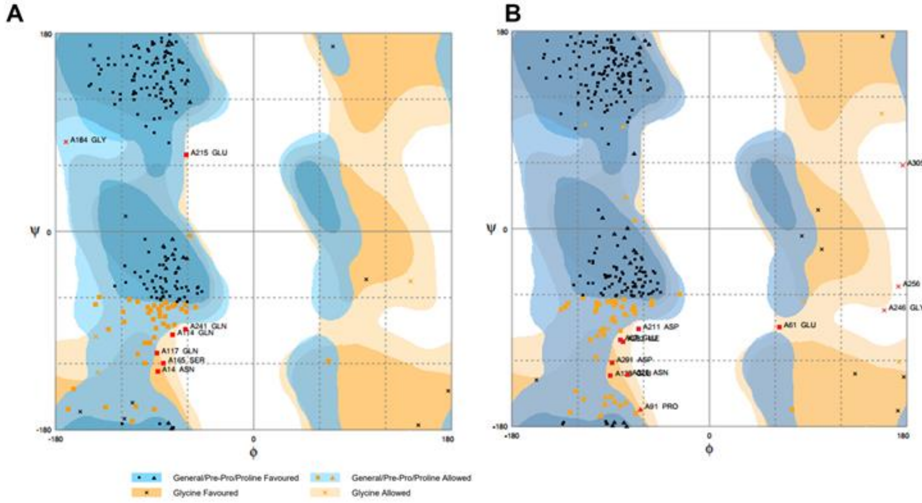

S4 Figure. Ramachandran plot analysis after 200 ns of MD simulation. (A) MAGI-I 255. (B) MAGI-1 329.

Table S1. Comparison of types and number of interactions between E6, its variants and our models of MAGI-1. Hydrogen bonds, Salt bridges, Non-bonded contacts.

Complexes	Hydrogen bonds	Salt bridges	Non-bonded contacts
**E6R/MAGI-1 255**	**9**	**3**	**156**
**E-G350/MAGI-1 255**	**14**	**9**	**156**
**E-C188/G350 /MAGI-1 255**	**25**	**7**	**231**
**E-A176/G350/ MAGI-1 255**	**23**	**10**	**382**
**AAa/MAGI-1 255**	**17**	**3**	**239**
**AAc/MAGI-1 255**	**13**	**2**	**199**
**Complexes**	**Hydrogen bonds**	**Salt bridges**	**Non-bonded contacts**
**E6R/MAGI-1 329**	**11**	**6**	**156**
**E-G350/MAGI-1 329**	**18**	**4**	**220**
**E-C188/G350 /MAGI-1 329**	**23**	**8**	**298**
**E-A176/G350/ MAGI-1 329**	**18**	**5**	**212**
**AAa/MAGI-1 329**	**24**	**8**	**242**
**AAc/MAGI-1 329**	**12**	**6**	**189**

Table S2. Detailed results of amino acidic interaction between E6, its variants and our two MAGI-1 models.

E6 VPH-16	Residues	Residues MAGI1-1 255
**E6-referece**	**Y81, R10, C30, R77, V31, Y32, R55, Y76, C66, A61, F69, K65, V62, V53, L67, N127, C51, C80, R131, G130, Q107, I73, R129 and Y70.**	**G75, A234, Y74, T161, N163, V79, D80, W66, G65, A64, Y78, I76, H231, G230, Y77, E61, G70, K68 and E67.**
**E-G350**	**L72, R146, K68, N105, S143, E75, R135, S142, C139, S138, R144, C106, D44, E114, E113, M137, K121, R117, R141 and C140.**	**D57, S58, E59, L60, E61, P63, E55, L56, L62, A64, G65, Y78, K68, W66, D80, V79, E67 and H81.**
**E-C188/G350**	**R146, T145, K72, R144, R141, E150, S82, R147, E75, R124, R135, Y84, E148, Y79, H78, R77, Y76, F69, C82, R129, Y70, Y81, G130, N127,Y92 and R131.**	**S58, D57, L62, E67, L60, E59, P63, R162, A64, E61, L56, E54, E55, L167, S165, P164, N163, F160, K230, P193, F196, L229, D194, E70, K68, V221, N224, Q235, S222, C227, F171 and D225.**
**E-A176/G350**	**R146, Y84, N105, R124, R147, E148, S82, Y81, Q90, Q91, C33, T145, S143, R131, R129, S142, C80, G130, R77, I73, Y79, Y76, E75, K72, S74, S71, K68, D64, P69, K34, L67, K65, C66, C63, Y70, V62, R55, D56, Y32 and V53.**	**E55, A64, L167, N163, E54, P164, S165, R162, C227, D225, K85, T226, L229, L60, E61, P63, E70, E59, H231, P193, G230, D194, F159, F196, K68, F160, E67, V79, I69, Y78, H81, D80, R84, Y77, E151, N83, Y88, Y74, I82, E89, Q87, T86, E94, N90, P91 and R150.**
**AAa**	**R131, R29, S143, R10, Y32, Y70, F69, S74, R55, I73, Y76, Y78, R77, G130, Y92, K72, W132, N127, Y81, L151, E148, E75, Y79, R144, R124, S82, R147 and H126.**	**E67, W66, K238, E166, L62, P164, R162, L167, N163, F171, E59, S165, D194, T161, F196, F160, E195, I82, F159, K157, K68, C227, E70, I243, L229, P244, Q241, Q122, S242, G230, H231 and Q119.**

AAc	C80, R129, R135, H126, Y81, G130, Y79, Y78,Y76, R124, E75, S82, R77, Y84, V83, Y32, Y70, F69, Q91, K72 and K65.	V221, C227, N163, H231, F171, E252, L229, T161, P164, G230, R162, S165, F160, F196, D194, F159, I76, I82, L167, E70, K168, K68 and E67.
	**Residues**	**Residues MAGI-1 329**
E6-referece	D64, K72, K68, K65, Y76, R147, F69, I73, L50, R77, Y70, R129, H78, V62, Q17, L67, R131, C66, V31, D56, Y32, R55 and V53.	D57, E54, S58, K157, A64, L62, E65, T53, G65, W66, V79, Y78, H81, I82, E67, D80, V92, E94, N83, R97, P91, N90 and E89.
E-G350	R77, I73, R129, Y76, D56, H78, I128, Y70, S71, N105, R48, C66, L67, I104, Q107, D74, L50, R131, Y32, F69, K72, R55, V31, V53 and K34.	A64, W66,G65, Q106, Y78, I69, K68, E61, E67, V79, T86, R84, H81, I82, D80, L93, E94, V92, T53, E54, D57, E102, R97, Q105, L101 and Q110.
E-C188/G350	K72, E75, R77, Y76, S142, R144, M137, R117, S143, R141, C140, K68, K65, F69, D64, E148, R147, T145, T149, R146, L151 and Q150.	S269, L270, V284, N285, G286, E55, E59, V51, Q39, F261, D262, L60, V317, D316, T272, T26, N23, T24, R33, E280, P263, N36, D265, P41, H22, D264, L35, L42, N145, D21, E43, E151, T12, L25, Y11, A10, E13, E8, M9 and I20.
E-A176/G350	R147, Y32, R55, G57, Y70, I73, K72, F69, S71, S74, Q91, E75, I128, Y79, H126, Y76, H78, R77, V83, R129, R131, C80, S82, R124 and Y81.	E89, L62, Q87, D316, E61, K157, A64, Y78, T86, W66, G65, V79, E67, D80, D57, Q106, H81, N83, I82, R84, P91, T53, H52, E94, N90, E54, L93, V92, and R97.
AAa	R77, Y76, K72, E75, Y79, R135, S138, S143, R141, S142, L151, K121, R144, T145, Q150, R147, E148, T149 and R146.	L277, E321, D278, V317, A318, D316, A315, N320, L6, L154, S58, E61, E59, L60, P260, K200, L259, P153, V51, V188, D262, F261, D264, L35, P263, Q39, K40, C34, P41, L42, E43, H52, G50 and E55.
AAc	K65, K68, F69, Y78, R129, S74, R77, L50, V62, Y70, I73, R102, Q107, F45, L67, V53, Y32, Y76, Y60, K72, D56, C33, V31, K34 and R55.	T53, E54, S58, D57, L56, Y78, D80, I69, K68, E67, V79, H81, I82, E94, W66, A64, G65, N83, L62, R84, R97, K157, E61, L93, V92, L101, N90 and E89.
